# mRNA Vaccine Era—Mechanisms, Drug Platform and Clinical Prospection

**DOI:** 10.3390/ijms21186582

**Published:** 2020-09-09

**Authors:** Shuqin Xu, Kunpeng Yang, Rose Li, Lu Zhang

**Affiliations:** 1 State Key Laboratory of Genetic Engineering, Institute of Genetics, School of Life Science, Fudan University, Shanghai 200438, China; 18861830310@163.com (S.X.); yangkp0521@gmail.com (K.Y.); 2 M.B.B.S., School of Basic Medical Sciences, Peking University Health Science Center, Beijing 100191, China; 18121321336@163.com; 3 Shanghai Engineering Research Center of Industrial Microorganisms, Shanghai 200438, China

**Keywords:** mRNA, mRNA vaccine, in vitro transcription, self-adjuvanting property, delivery carriers, infectious diseases, cancer therapeutics

## Abstract

Messenger ribonucleic acid (mRNA)-based drugs, notably mRNA vaccines, have been widely proven as a promising treatment strategy in immune therapeutics. The extraordinary advantages associated with mRNA vaccines, including their high efficacy, a relatively low severity of side effects, and low attainment costs, have enabled them to become prevalent in pre-clinical and clinical trials against various infectious diseases and cancers. Recent technological advancements have alleviated some issues that hinder mRNA vaccine development, such as low efficiency that exist in both gene translation and in vivo deliveries. mRNA immunogenicity can also be greatly adjusted as a result of upgraded technologies. In this review, we have summarized details regarding the optimization of mRNA vaccines, and the underlying biological mechanisms of this form of vaccines. Applications of mRNA vaccines in some infectious diseases and cancers are introduced. It also includes our prospections for mRNA vaccine applications in diseases caused by bacterial pathogens, such as tuberculosis. At the same time, some suggestions for future mRNA vaccine development about storage methods, safety concerns, and personalized vaccine synthesis can be found in the context.

## 1. Introduction

**Figure 1:**
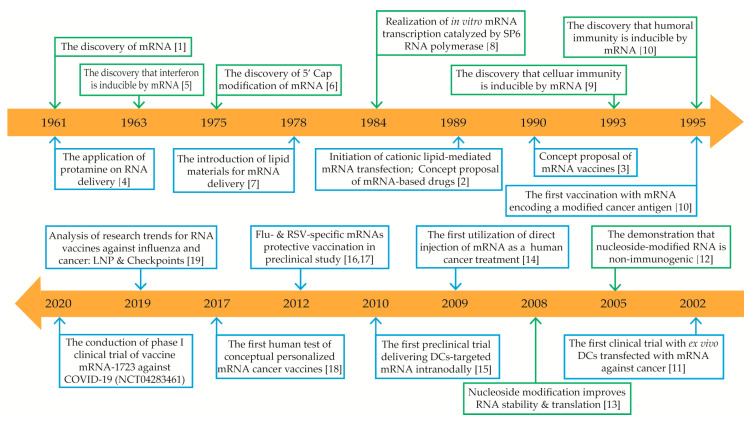
Timeline of some key discoveries and advances in the development of mRNA-based drug technology. Green boxes represent discoveries and advances in mRNA mechanisms; blue boxes represent discoveries and advances in mRNA-based drug applications. Abbreviations: mRNA, messenger RNA; 5′ cap, five-prime cap; LNP, lipid nanoparticles; COVID-19, coronavirus disease 2019; DCs, dendritic cells.

mRNA, an intermediate hereditary substance in the central dogma, was first discovered in 1961 by Brenner et al. [1]. However, the concept of mRNA-based drugs was not conceived until 1989, when Malone et al. demonstrated that mRNA could be successfully transfected and expressed in various of eukaryotic cells under the package of a cationic lipid (N-[1-(2,3-dioleyloxy) propyl]-N,N,N-trimethylammonium chloride (DOTMA)) [2]. In 1990, in vitro-transcribed mRNA was sufficiently expressed in mouse skeletal muscle cells through direct injection, which became the first successful attempt on mRNA in vivo expression and thus proved the feasibility of mRNA vaccine development [3]. Since then, mRNA structure researches and other related technologies have been rapidly developed. Under this condition, several development restrictions stemmed from mRNA instability, high innate immunogenicity, and inefficient in vivo delivery have been mitigated, and now mRNA vaccines have been widely studied in different kinds of diseases (Figure 1) [1,2,3,4,5,6,7,8,9,10,11,12,13,14,15,16,17,18,19].

mRNA vaccines have demonstrated many specific advantages that conventional vaccines do not have. First of all, mRNA can theoretically meet all genetic information requirements to encode and express all kinds of proteins. Vaccine developing efficiency can be optimized by modifying mRNA sequence, which is a more convenient way compared to other kinds of vaccine modification [20,21]. Furthermore, most of the mRNA vaccine production and purification processes are quite similar despite different encoded antigens, so it is potential to be retained or even standardized to develop other similar mRNA vaccines [20,22]. Utilizing in vitro transcription also makes mRNA vaccines production easier [20,21,22]. Accordingly, it is obvious that mRNA vaccines can save both time and economic costs. Second of all, mRNA has self-adjuvanting properties which activate strong and long-lasting adaptive immune responses through tumor necrosis factor-α (TNF-α), interferon-α (IFN-α) and other cytokines secretion by immune cells [23], while polypeptide and protein based vaccines need extra adjuvants to achieve a similar goal [24]. The in vivo expression of mRNA can also avoid protein and virus-derived contamination [20]. By modifying the mRNA sequence and delivery system, the expression activity and in vivo half-life of mRNA can be effectively regulated [19,21,24]. Thirdly, in comparison with DNA-based vaccines, mRNA vaccines can express target proteins more efficiently because of their expression in the cytoplasm without entering the nucleus [25]. In addition, due to the chemical constitution of the mRNA sequence, which is different from DNA constitution and lack of CpG islands, there is a lower possibility for mRNA to integrate into host DNA genome and induce a smaller immune rejection reaction [25]. Besides, mRNA is only transiently active, making it easy to be completely decomposed via physiological metabolic pathways; therefore, it would not act as a burden to the host homeostasis [25].

After the first mRNA-based drug company was established in 1997, a large number of groups began to research and develop mRNA-based drugs [25]. So far, over twenty mRNA-based candidate drugs have entered the clinical trial stage. The market value for the mRNA vaccine field has also increased, reaching up to tens of billions of dollars, which signifies a broad prospect for mRNA-based drugs development, especially mRNA vaccines. In particular, mRNA vaccines have a huge potential on rapidly responding to emerging epidemics, e.g., the global explosion of the coronavirus disease 2019 (COVID-19), stimulating more interest and research expectations from worldwide scientists [26,27].

## 2. mRNA In Vitro Synthesis and Modification

To date, in vitro transcription technology of mRNA has been mature, and the most popular method is using T3, T7, or SP6 RNA polymerase and linear DNA (linearized plasmid DNA or synthetic DNA prepared by PCR) for mRNA synthesis. There are some basic structural elements of mature mRNA in the eukaryocyte that are required to keep mRNA functional, including five-prime cap (5′ cap), five-prime untranslated region (5′ UTR), open reading frame (ORF) region, three-prime untranslated region (3′ UTR), and poly (A) tail structure [21,28]. Keeping mRNA structure intact is beneficial for mRNA stability and expression capability. Modifying the mRNA sequence based on its complete structure can further optimize the efficiency of an mRNA vaccine. However, the initial product of mRNA in vitro transcription is the mixture of targeted mRNA, untargeted RNA, nucleotides, oligodeoxynucleotides, and proteins [20]. To purify the mRNA, precipitation and extraction techniques are used to remove common impurities and chromatographic techniques are generally used to separate the target mRNA from other mRNA impurities in this system [29].

### 2.1. Five-Prime Cap (5′ cap) and Modification

mRNAs from the eukaryotic and partial viral genomes have a 7-methylguanosine (m^7^G) cap at the 5′ end of the mRNA sequence (m^7^GpppN structure), which connect to the first RNA nucleotide through a 5′, 5′-triphosphate bridge (ppp) during mRNA in vitro transcription. The 5′ cap can eliminate free phosphate groups in the mRNA sequence so as to significantly enhance the stability of mRNA, which allows the ribosome to recognize the beginning of mRNA and improves translation efficiency by binding to the eukaryotic translation initiation factor 4E (eIF4E) [25,30]. So it is obvious that 5′ cap modification can be crucial to mRNA property improvement. There are two common approaches in terms of in vitro mRNA capping. Firstly, adding a regular cap analog, m^7^GpppG structure, to the mRNA transcription system can achieve mRNA capping along with in vitro transcription [20,25]. Secondly, mRNA capping can also be completed by capping enzyme reaction after the initial in vitro transcription [25,31].

Capping with cap analog is the most common capping method of mRNA in vitro transcription, but studies have found that regular cap analog can reversely bind to the mRNA sequence [32]. In this case, mRNA isomers are formed and lead to low efficiency of mRNA downstream translation. To avoid reverse incorporation of 5′ cap, anti-reverse cap analogs (ARCA) have been developed [32,33]. ARCA is modified at the C2 or C3 position to ensure that the methyl groups react with the hydroxyl groups at the correct site during transcription. Compared to regular cap analog, ARCA-capped mRNA has a higher translation efficiency [32,33,34]. In recent years, further modification on the ARCA structure has been developed to improve mRNA properties. Phosphorothioate modifying based on ARCA, for example, would enhance the translation efficiency of mRNA by increasing its affinity for eIF4E, and has the ability to decrease the susceptibility to decapping enzymes so as to improve the mRNA stability [35,36,37]. Kuhn et al. showed that m_2_^7,2′-O^Gpp_S_pG (β-S-ARCA) could significantly enhance the stability and translation efficiency of mRNA in immature dendritic cells (DCs) [35]. In 2016, Strenkowska et al. synthesized cap analogs that were composed with 1,2-dithiodiphosphate modification, ARCA, and an extended polyphosphate chain, named “2S analogs”, the benefits of which enabled 2S analogs to function better than any S-ARCA used in clinical trials [38]. Another cap analog, a co-transcriptional capping method called “CleanCap,” was developed in 2018 [39]. It utilized an initiating capped trimer to yield a naturally occurring 5′ cap structure, which increased the capping efficiency to nearly 90–99% [22,39].

### 2.2. Optimization of Untranslated Regions (UTRs)

UTRs are non-coding parts of mRNA sequence located at the upstream (5′ UTR) and downstream (3′ UTR) domains of the mRNA coding region. As reported, UTRs are related to mRNA replication and translation processes, and they can greatly alter mRNA decay and translation efficiency through reactions with RNA binding proteins [20,22]. In an attempt to enhance mRNA stability and translation efficiency, it is essential to ensure the optimization of UTRs.

Generally speaking, UTR optimization is to increase the in vivo mRNA expression level. For instance, the widely-used 3′ UTR sequence derived from α-globin and β-globin contains translation and stability regulatory elements [30]. 3′ UTR is normally considered to be a concentrated region full of unstable factors in mRNA, so averting unstable sequences while synthesizing 3′ UTR can increase mRNA stability. AU-enriched sequences and GU-enriched sequences are related examples of this [40,41]. On the other hand, introducing stable elements to 3′ UTR can also significantly improve the stability of mRNA and expand its half-life [42,43]. Orlandini von Niessen et al. once connected two random 3′ UTRs which contained stable elements in series, and successfully improved the translation efficiency of mRNA [43].

5′ UTR directly affects the translation of its downstream sequence ORF, so the optimization of 5′ UTR should not influence the normal translation process of the ORF. Avoiding the gene sequence in 5′ UTR, which is identical to the upstream of ORF, can effectively prevent false start and replacement of the reading frame during mRNA translation [44]. Additionally, some particular sequences can be added to 5′ UTR to enhance the stability of mRNA and the accuracy of translation. For example, Kozak et al. inserted sequence GCC-(A/G)-CCAUGG in this region, leading to a more accurate start of translation process [45]. Study also shows that over-stabilized secondary structure of 5′ UTR would hinder the binding of ribosomes to mRNA, and short and loose 5′ UTR is more conducive to the mRNA translation processes [46].

### 2.3. Codon Optimization of Open Reading Frame (ORF)

As the coding region of mRNA, the translatable rate of ORF region is definitely crucial. Therefore, choosing the appropriate codons in this region can optimize the overall translation efficiency of mRNA. Optimized ORF sequence usually incorporates synonymous frequent codons and/or codons with higher tRNA abundance to replace rare codons in ORF, so that highly expressed genes can be translated using the same codons of the host and/or guaranteed the ampleness of tRNA during the expression of exogenous mRNA [47]. However, high translation rate of mRNA is not all beneficial, as some proteins require a low translation rate to fold correctly, stably, and effectively; in this case, using codons with low frequency in ORF can yield protein products of higher quality [22]. Therefore, for different antigens, we should use different codon optimization strategies to improve mRNA translation rate and ensure the expressed antigen quality at the same time.

### 2.4. Poly (A) Tail and mRNA Stability

Poly (A) tail and the 5′ Cap structures are both crucial elements during mRNA translation. Poly (A) sequence can slow down the degradation process of RNA exonuclease, which increases stability, extends in vivo half-life, and enhances translation efficiency of mRNA [22]. Moreover, Poly (A) binding protein (PABP) can link to the 5′ Cap through translational initiation factors, such as eIF4G and eIF4E, which in turn affects the closed-loop structure of mRNA and synergistically regulates the stability and translation efficiency of mRNA [22,48,49]. However, PABP can also bind to adenylation complexes and participate in translation inhibition process mediated by microRNA [49]. The contradictory function of PABP indicates that various Poly (A) sequence length can affect mRNA translation efficiency differently. There are different methods to synthesize a Poly (A) structure, among them, in vitro transcription process with DNA template with Poly (A) structure information can yield a defined Poly (A) sequence length [25]. Recombinant Poly (A) polymerase can also be used to add Poly (A) structures by undergoing an enzymatic polyadenylation after initial mRNA transcription, in which case Poly (A) structural mixtures of different lengths can be obtained [25]. Early studies suggest that a long Poly (A) sequence can improve mRNA stability. For example, the optimal length of Poly (A) sequence in DCs is roughly between 120–150 nucleotides [25,50], and over 300 nucleotides of Poly (A) sequence length in human primary T cells can become more conducive in increasing mRNA stability and translation efficiency [51]. When Poly (A) sequence length is less than 20 nucleotides, it would reduce mRNA translation efficiency [52]. However, in 2017, Lima et al. found that mRNAs with high translation efficiency generally had short Poly (A) sequences through novel genome-wide research techniques, whilst short Poly (A) structures were generally found in well-translated eukaryotic mRNAs [49]. Therefore, it has been indicated that since the lengths of Poly (A) sequences required for high translation efficiency mRNA in various types of cells are different, adjustments should be made to optimize the translation efficiency of mRNA.

## 3. Immunogenic Regulation of mRNA

**Figure 2:**
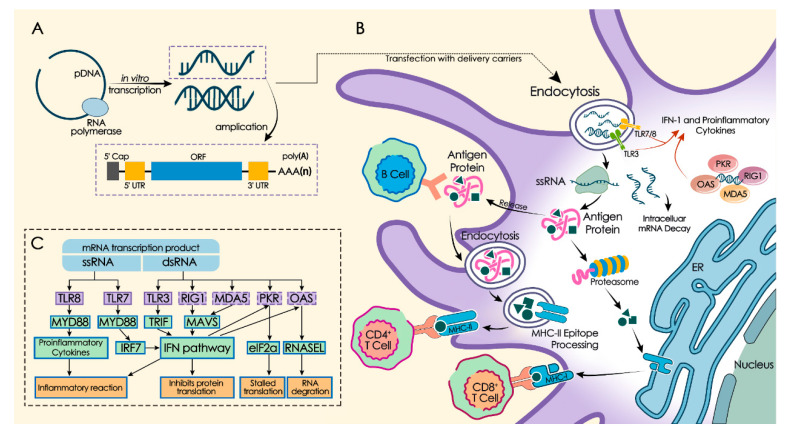
mRNA in vitro transcription and innate immunity activation. (**A**) mRNA in vitro transcription. Using DNA with the antigen-encoding sequence as template, mRNA in vitro transcription products contain single-stranded RNA (ssRNA), double-stranded RNA (dsRNA), etc. The ssRNA structure normally includes five-prime cap (5′ cap), five-prime untranslated region (5′ UTR), open reading frame (ORF) region, three-prime untranslated region (3′ UTR), and poly (A) tail structure. (**B**) RNA translation and antigen presentation. Through endocytosis, mRNAs enter the cytoplasm. Some mRNAs combine with ribosomes of the host cell and translate successfully. Antigen proteins can be degraded to antigenic peptides by proteasome in the cytoplasm and presented to cytotoxic T lymphocytes (CTLs) via major histocompatibility complex (MHC) I pathway. Or, they can be released out of the host cell and taken up by DCs. Then, they are degraded and presented to helper T cells and B cells via MHC-II pathway. B cells can also recognize released antigen proteins. (**C**) Self-adjuvant effect. Various of pattern recognition receptors (PRRs) can recognize mRNA in vitro transcription product. ssRNA can be recognized by endosomal innate immune receptors (e.g., Toll-like receptor 7 (TLR7), TLR8). dsRNA can be recognized by endosomal innate immune receptors (e.g., TLR3) and cytoplasmic innate immune receptors (e.g., protein kinase RNA-activated (PKR), retinoic acid-indu [21] cible gene I protein (RIG-I), melanoma differentiation-associated protein 5 (MDA5), and 2′-5′-oligoadenylate synthase (OAS). Based on those, mRNA products can stimulate the secretion of pro-inflammatory cytokines and type I interferon (IFN), which leads to antigen-presenting cells (APCs) activation and inflammatory reaction. However, they can also activate antiviral enzymes that cause stalled mRNA translation and mRNA degradation.

Based on its self-adjuvanting effect, mRNA can exhibit some properties similar to the mRNA virus when it works as the vector of exogenous genes. In this case, mRNA can be recognized by antigen-presenting cells (APCs), which subsequently activates pattern recognition receptors (PRRs) such as Toll-like receptor 3 (TLR3), TLR7, and TLR8 [30,53,54]. The double-stranded RNA (dsRNA) can combine with some Retinoic-acid-inducible gene I (RIG-I) -like receptors (RLRs) in the cytoplasm, such as RIG-I and melanoma differentiation-associated 5 (MDA5), which promotes APCs maturation, pro-inflammatory cytokines secretion, and type I interferon (IFN) secretion [55,56]. Eventually this leads to strong antigen-specific humoral and cellular immune responses (Figure 2). However, subunit vaccines composed of peptide or protein antigens are generally unable to activate PRRs, so it is necessary to add adjuvants which can initiate and support adaptive immune responses, achieving the final result of carrying out the body’s immune response of subunit vaccines [25]. Therefore, mRNA’s strong adaptive immune response and self-adjuvanting property can provide a huge advantage shown in mRNA vaccines. Single-stranded RNA (ssRNA) can trigger the DCs’ antiviral activation state through TLR7 and TLR8 recognition during mRNA in vivo transmission [57]. The dsRNA contaminants can also trigger immune activation via TLR3 recognition [19,20]. However, excessive immune response stimulated by mRNA in the cytoplasm would stimulate cells to secrete large amounts of type I IFN and other interferons which can inhibit the translation of mRNA and eventually lead to translational stagnation, RNA degradation, CD8 (cluster of differentiation 8)^+^ T cells activation reduction, and ultimately immune response termination [13,21,58]. This could leave negative effects on some mRNA applications such as vaccines and protein replacement therapies. Self-adjuvanting properties of mRNA have both advantages and disadvantages in mRNA vaccine applications, therefore, it is necessary to form mRNA immunogenic regulations according to different medical demands, which in return would effectively improve the application efficacy of mRNA vaccines.

### 3.1. mRNA Purification Modulates Self-Adjuvanting Property

mRNA in vitro transcription product often contains dsRNA contaminants. dsRNA, which is a simulant of RNA virus genome replication intermediates, can promote type I IFN production [20,55]. Therefore, the purification of an mRNA in vitro synthetic product can effectively reduce type I IFN immune response of mRNA vaccines and increase mRNA translation efficiency [21]. Studies have shown that chromatographic methods (fast protein liquid chromatography, high-performance liquid chromatography, etc.) can effectively remove dsRNA from mRNA products; after purification, the mRNA translation level in primary cells can be increased by 10–1000 times while the cytokine secretion level still remains relatively high [29,59].

### 3.2. Optimization of mRNA Sequence to Regulate Self-Adjuvanting Property

ssRNA can also work as a potent pathogen-associated molecular pattern (PAMP) that elicits a strong immune response and stimulates type I IFN production. Type I IFN can induce numerous types of IFN-stimulated genes (ISGs) to inhibit mRNA translation [22]. For instance, IFN-inducible protein with tetratricoid repeats (IFIT) can combine with the 5′ Cap structure or interact with eIF3 to disrupt the mRNA translation process [22,60]. Therefore, optimizing mRNA sequence can regulate the ability to activate the immune response of mRNA vaccines [21,58,61].

PRRs can recognize Cap0 (m^7^GpppN)-capped or uncapped mRNA and inhibit its translation [62]. In 2014, Kumar et al. evaluated the ability of PRRs to recognize three forms of capped mRNA, including Cap0-capped, Cap1 (m^7^GpppNmN)-capped, and uncapped mRNA. They discovered that Cap1-capped mRNA was still translated after being recognized by PRRs, while Cap0-capped and uncapped mRNA were not [60]. Therefore, choosing appropriate 5′ Cap structure can avoid excessive immunity response.

Modification of the ORF region can also reduce the strong immune response caused by PRRs recognition, and enhance the translation level of mRNA [22]. In 2011, Anderson et al. studied the difference between unmodified mRNA and pseudouridine modified mRNA [63]. The ability of mRNA to be recognized by 2′-5′-oligoadenylate synthetase (OAS protein, induced by type I IFN) and mRNA stability were assessed, and results showed that the pseudouridine modified mRNA had lower efficiency in terms of OAS activation, lower rate of RNA degradation, and higher efficiency of mRNA translation [63]. Karikó et al. intravenously injected pseudouridine modified mRNA in mice, and found out that there was a higher target protein expression in the spleen and lower IFN-α concentration in serum compared with unmodified mRNA treatment [13]. Uracil analog is the most common analog used in mRNA modification, and some other base analogs can also be used for mRNA sequence modification. Kormann et al. and Mays et al. used different rates of 5-methyl-cytidine and 2-thiouridine to modify mRNA sequence, in which both effectively reduced the recognition rate of PRRs, and increased mRNA intracellular stability [64,65].

### 3.3. Adding Adjuvants to Optimize mRNA Immunogenicity

Some studies need the enhancement of the immunogenicity of mRNA vaccines and adding adjuvants to the mRNA vaccine system can meet this requirement. Formulation of self-amplified RNA vaccines with the traditional adjuvant MF59 (made by Novartis) and cationic nanoemulsion (CNE) have proven to enhance the immunogenicity and efficacy of mRNA vaccines in various animal models [21,66]. Certain immunomodulatory molecules also have adjuvant activity. TriMix, a new adjuvant strategy developed by Vrije Universiteit Brussel, consists of mRNAs that encode three immune activator proteins—CD70, CD40 ligand (CD40L) and constitutively active TLR4 [53,67,68]. TriMix mRNA can increase the immunogenicity of naked, unmodified, unpurified mRNA, and it is also related to the enhancement of DCs’ maturation and cytotoxic T lymphocyte response [67]. In 2018, Leal et al. adopted the TriMix naked mRNA strategy to treat acquired immune deficiency syndrome (AIDS) patients. Treatment using high doses of TriMix mRNA showed that a high human immunodeficiency virus (HIV)-specific T cell response could be stimulated and detected [69]. The high safety and tolerability of this strategy has been demonstrated in this research [69].

Some mRNA delivery vehicles can also increase the adjuvant effect, such as cationic lipid and protamine. In 2013, researchers used the mRNA vaccine immunization strategy with cationic lipid 1,2-dioleoyl-3trimethylammonium-propane/1,2-dioleoyl-sn-glycero-3-phosphoethanolamine (DOTAP/DOPE) as the assigned adjuvant, and stimulated more pro-inflammatory cytokines and type I IFN secretion than naked mRNA in DCs [61]. After subcutaneous injection of this mRNA vaccine in mice, large amount of type I IFN secretion and rapid aggregation of inflammatory monocytes could be detected in lymph nodes transiently [61]. This indicates that cationic lipids can strengthen the adjuvant effect and the efficacy of mRNA vaccines to a certain extent [24,70]. Researches also demonstrated that mRNA and protamine complexes could act as danger signal and elicit T-help 1 cell (Th1) responses via TLR7 and TLR8 involving [14,71]. The RNActive^®^ vaccine platform designed by CureVac used co-delivered RNA and protamine complex as the adjuvant to induce Th1 T cell responses, and naked, unmodified, and sequence-optimized mRNA as the antigen to develop mRNA vaccines [54]. In this technique, protamine-formulated RNA only works as an adjuvant, not as a mRNA carrier, enabling more RNActive^®^ vaccines to arouse strong immune responses in many pre-clinical models, which can successfully prevent attacks from various influenza strains [21,54]. Kowalczyk et al. revealed that RNActive^®^ vaccine treatment in mice could initiate a balanced and strong specific immune response with intradermal immunization [72]. This immune stimulation only existed in the stimulated site and lymphoid organs, and no pro-inflammatory factors were detected in serum. Overall, RNActive^®^ technology is a new effective technique of mRNA vaccine with high levels of safety and flexibility.

## 4. mRNA Delivery System

mRNA needs to enter the host cytoplasm to express specific antigens to remain functional; however, the mRNA molecule is not small enough to pass through cell membrane by free diffusion [21,25]. Additionally, mRNA and cell membrane are both negatively charged, which increases the difficulty of mRNA delivery. Furthermore, mRNA can be easily degraded by extracellular ribonucleases which exist in skin and blood [21,25]. Therefore, delivering mRNA into enough numbers of cells with sufficiently high translation levels is one of the most difficult application problems of mRNA vaccines, as it demands highly specific and efficient mRNA delivery systems [73,74]. A variety of mRNA delivery methods and mRNA delivery vehicles have been developed and applied currently (Table 1).

### 4.1. Naked mRNA Delivery System

#### 4.1.1. Direct Injection of Naked mRNA

Early study has demonstrated that naked mRNA in vivo injection can provoke the immunotherapy response in mice [3]. At present, administration strategies of mRNA generally include subcutaneous injection, intradermal injection, intranodular injection, intramuscular injection, intravenous injection, intratumoral injection, etc., which are essential methods that help stimulate antigen presentation and initiate immune responses [21,117,118]. In 2013, Phua et al. discovered that delivery efficiency of subcutaneous injection of naked mRNA in mice was even higher than mRNA nanoparticle delivery methods [78]. Van Lint et al. suggested that intratumoral injection of tumor-associated mRNA would elicit an appropriate immune response and believed that it could be a promising vaccination strategy for the impending future [67]. These days, direct injection of naked mRNA is mainly used to treat or prevent infectious diseases [97]. However, even though the injection of naked mRNA can cause immune response, the working effect of this delivery method is relatively weak, and the naked mRNA is often rapidly degraded after injection. Direct injection of naked mRNA is too simple and primitive to be applied in human patients, and it is often used as an administration route to inject modified mRNA vaccines with other delivery systems to achieve better vaccine effects.

#### 4.1.2. Physical Delivery of Naked mRNA

The efficiency of naked mRNA antigen presentation can be improved with the assistance of common physical methods including electroporation, gene gun, microneedles, etc. [119]. Electroporation can increase mRNA delivery efficiency without the demand of other mode receptors, which can reduce unnecessary immunoreactions [120]. Electroporation also has an adjuvant effect that it can recruit pro-inflammatory cells and induce the production of cytokines at the inoculation site, improving the immunogenicity of mRNA [119]. In 1987, Callis et al. found that electroporation could be used to transfer mRNA into animal and plant cells with low transfection efficiency [121]. However, the target intracellular expression product was high enough to reach the detection level. In 2017, the mRNA transfection efficiency in DCs had reached 50–90% for electroporation method [18]. The gene gun method, using compressed helium gas as an acceleration force to push mRNA coated on the surface of gold particles into host cells, is an efficient method of mRNA delivery [119]. In 1996, Qiu et al. used gene gun method to transfer the human alpha-1 antitrypsin mRNA into the mouse skin and successfully triggered the antibody response [122]. Peking et al. developed a mRNA-based therapy for genetic skin diseases restoration, mRNA was effectively transported to the target skin layers in mice by gene gun delivery [83]. Despite its advancements, the gene gun method is rarely used in large animals and humans. Physical ways to deliver mRNA may affect the physiological structure and activity of cells, even causing abnormal cell death. Therefore, applying physical mRNA deliveries in human is potentially hazardous [21,119].

### 4.2. Ex Vivo Loading of DCs Delivery System

DCs are one of the most potent APCs of immune system. They can present processed antigens to CD4^+^, CD8^+^ T cell via the major histocompatibility complex (MHC), which triggers cellular immunity [21,123]. Meanwhile, DCs can also present intact antigens to B cells, triggering humoral immunity [124]. The common way to use DCs as mRNA delivery vehicles is to transfect mRNAs encoding peptides, proteins or other antigens into DCs via in vitro, and then transfer the processed DCs back into the host body to start the antigen-specific immune response [125]. The DCs-mRNA delivery system does not need to be combined with other carrier molecules and can generate high delivery efficiency. In this context, this delivery system is widely used in pre-clinical experiments, animal models and clinical researches [87,88,126,127]. Moreover, this strategy has been mainly applied in cancer treatment because the elicitation of cellular immune response is predominant [128]. However, the mRNA transfection rate is quite low if only by DCs endocytosis, and electroporation method is often used to further improve the mRNA transfection rate [129]. Gay et al. used electroporation to transfer the mRNA encoding HIV antigens into DCs for HIV treatment, after intradermal injection, the number of HIV-specific CD28^+^/ CD45RA-CD8^+^ factors/cytotoxic T-lymphocytes (CTLs) was at least 2 times higher than control, which enhanced the T cell immunological reactions of HIV patients [129]. Another unignorable barrier to clinical application of ex vivo-loaded DC mRNA vaccines is that time- and money-consuming production process cannot meet the huge quantity demand of mRNA vaccine for some treatments. Besides, the immune response caused within several hours after mRNA transfection can be lost during the time-consuming in vitro preparation process, leading to reduction of the therapeutic effect of mRNA vaccines [19]. Out of these considerations, diseases that require large amounts of mRNA vaccine treatment in the short term should give preference to delivery systems with a fast production speed. Delivery systems that are able to directly target mRNA to in vivo APCs can also be considered.

### 4.3. Protamine-Formulated Delivery System

Protamine is an alkali cationic protein with resin-like structure. Combining mRNA with protamine in different mass ratios can yield electrostatic protamine-mRNA complex particles with different diameters [54]. This tight conjugate form can effectively protect mRNA from being degraded by serum RNases, and the complex can cause a strong immune-reaction of immune cells such as DCs, monocytes, B cells, natural killer cells, and neutrophils [14,71,72]. This indicates that protamine has the potential to be used not only as a mRNA carrier, but also as an immune activator. In 1961, protamine was already studied as one of the first delivery materials for long RNA [130]. Fotin-Mleczek et al. used protamine as the delivery material during the vaccination of mRNA tumor vaccine, and successfully elicited a complete specific anti-tumor response [131]. When the mass ratio of protamine to mRNA is 1:2, the size of the electrostatic complex formed is about 300 nm, which is relatively stable and produces strong immune stimulation and high cytokine levels, with the downside of inhibiting protein expression significantly [131]. However, when the mass ratio of protamine to mRNA is 1:4, compared to the previous mass ratio of 1:2, the protein expression increased but the cytokine level decreased [131]. Hence, a common idea is that the mRNA translation efficiency and immune strength are limited in the protamine-formulated mRNA delivery system. And it is speculated that this defect may be related to the extremely tight electrostatic complex [20,131]. In recent years, protamine-formulated mRNA delivery system has been widely used in clinical trials, and gained pretty good clinical treatment effects, such as rabies, non-small cell lung cancer, etc. [90,91,92]. The RNActive^®^ vaccine platform, which use the protamine-mRNA only to activate immune responses, is a prevailing technique to resolve this problem [54]. Furthermore, to use protamine as a mRNA delivery and immune activator at the same time, structural optimization of protamine or searching for proteins similar to protamine in property as substitutes deserves our attention.

### 4.4. Cationic Lipid-Based Delivery System

As a commonly used gene carrier, cationic liposomes can also combine with negatively charged nucleic acids to form electrostatic complexes, improving mRNA delivery efficiency [132]. The cationic lipid-mRNA complex and other preparations together can form an 80–200 nm nanoparticle called lipid nanoparticles (LNP), which can be transfected into the cytoplasm by endocytosis. LNP is one of the most advanced mRNA delivery systems. This stable particle consists of ionizable cationic lipids, natural phospholipids, cholesterol and polyethylene glycol (PEG) [119]. The ionizable cationic lipid can promote the autonomous aggregation of mRNAs to form a ~100 nm particle and release mRNAs in the cytoplasm through ionization; natural phospholipids support the nanoparticles to form a lipid bilayer structure; cholesterol is used as a stabilizer to increase LNP stability; and PEG can extend the half-life of LNP complex [21,133]. mRNA is carried in the core of LNP which can be protected from degradation, and the lipophilicity property of LNP material allows the mRNA delivery complex to fuse with the host cell membrane and deliver mRNA into the cells by endocytosis [19,119]. LNP is often used as a short interfering RNA (siRNA) delivery system in early researches [134]. Nowadays LNP is also widely used in mRNA delivery processes. Geall et al. used LNP to deliver self-amplified RNA vaccines, which caused the mRNA expression level in mice to be significantly higher than that of naked mRNA, CD4 ^+^, and CD8 ^+^ T cell immune responses were also effectively induced. With different administration strategies, the immune-stimulation area provoked by LNP-mRNA can be different [17], and may achieve the targeted therapy need of different diseases. Pardi et al. found that injecting LNP-mRNA with the appropriate dose by subcutaneous, intramuscular, and intradermal methods could mediate local gene product expression [93]. LNP-mRNA treatment with intravenous injection, intraperitoneal injection, tracheal inhalation, etc. could achieve systemic expression of gene products, as reported in 2018, out of which intravenous injection showed the highest mRNA delivery efficiency, and the target protein products were successfully expressed in the liver for 4 days [101]. But it is notable that escape mechanisms of mRNA from complexes to free state for function in the cytoplasm are still incompletely understood. Change of ionization state of lipids with in vivo environmental pH is thought to be critical to the escape process [135]. Meanwhile, further research about the toxicity reduction and immunogenicity regulation of cationic lipid-based delivery system are also urgently needed.

### 4.5. Polymer-Based Delivery System

Currently, cationic polymers have been widely used as mRNA delivery vectors [133,136]. Commonly used polymer delivery materials include polyethylenimine (PEI), poly (beta-amino esters) (PBAEs), etc. Among them, PEI is one of the most widely used materials. PEI is a kind of cationic water-soluble polymer with either dendritic, linear, or branching structure, mainly used as a DNA/mRNA carrier [109,137]. There is a commercial linear PEI derivative called jetPEI ™, which was once used for DNA and siRNA transfection, and currently available for mRNA transfection [133,138]. However, PEI is also qualified with certain cytotoxicity that is hard to be degraded, so researchers often use fatty chains to modify low-molecule-weight PEI for the intention of reducing PEI toxicity [53,109,139]. PBAEs are biodegradable polymers originally developed for DNA transfection [140]. A study in 2007 showed that PBAEs could be used to deliver mRNA, and higher levels of mRNA transfection in vitro could be achieved when there is no serum protein in the system [141]. This research has led to the development and application of a variety of PBAEs that enhanced serum stability in vivo. There are now thousands of chemically different PBAEs created thanks to the simple synthetic method of PBAEs [133,142,143]. In addition, PBAEs and lipids can be formulated together to improve their serum stability. In 2016, Kaczmarek et al. developed a polymer-based delivery system by formulating PBAEs and lipid-PEG together, which had high serum stability and mRNA delivery efficacy and successfully detected the target mRNA product in the lungs of mice specifically by intravenous injection treatment [107]. Polymer-based materials are crucial competitors against lipids in mRNA therapeutics. Their toxicity, similar to cationic lipids, has been also thwarted them for broader application [135]. Apart from modification with other materials to improve the properties of polymer-based vectors, optimization for both molecular weight and branch pattern also seems to be a dependable direction.

## 5. Applications of mRNA as a Drug Platform

Immunotherapy, especially vaccines against infectious diseases and cancers, is the core field of the mRNA drug platform. Investigations of other areas such as reprogramming of cell fates and genome editing based on mRNA have been extensively reviewed [25,53], therefore they are not a subject of concern in this review. mRNA vaccines are generally categorized into two major types according to their construction and replication abilities: self-amplifying mRNA (SAM) vaccines and non-replicating mRNA vaccines.

The SAM vaccines are developed from an alphavirus genome with its gene encoding structural proteins replaced by the sequence encoding our wanted antigen, enabling intracellular RNA amplification, and abundant protein expression of the wanted antigen owing to the integrity of viral replication machinery [144]. The full length of naked SAM can be up to 9~10 kb. Due to self-replication, a remarkable low dose of this vaccine promises a huge amount of antigen production with a considerable duration of effectiveness (up to 2 months) [53]. The inoculation of SAM vaccines can make a simulation of the infection of acute pathogens owing to its PAMP, the replication of the self-adjuvanted antigen-encoding RNA and the protein expression occurring hours after the vaccination [145]. This property of SAM vaccines, nevertheless, remains controversial since it has the potential to limit the size of antigen-encoding sequence that can be accommodated, to affect the accurate regulation of induced inflammatory responses and even to elicit immune responses of the organism against those RNA replication factors, thus reducing the in vivo repeated efficacy of the vaccine [21].

Non-replicating mRNA vaccines have the complete structure of mature mRNA which contains the ORF segment that encodes our desired antigen. Owing to their small length (2~3 kb), there is no size restriction for the carrier capacity on the antigen, allowing better control of triggered immune responses as well as developing more affordable approaches from synthesis to storage [21,53]. Non-replicating mRNA vaccines have a huge potential to become the major cure for the current epidemic outbreak.

As mentioned earlier, studies regarding mRNA vaccines have largely completed concept establishment and initial exploration in the 1990s. In 1993, Martinon et al. successfully achieved in vivo induction of specific anti-influenza CTLs by intravenous or subcutaneous injection of mice with liposome-entrapped mRNA encoding influenza virus nuclear proteins, which was a pioneering mRNA vaccine vector attempt [9]. In 1998, Mandl et al. used the gene gun to deliver in vitro synthesized infectious RNA from a flavivirus, demonstrating induced protective immunity in mice by less than 1 ng of RNA [146]. Boczkowski et al. in 1996 obtained DCs with enhanced ability of antigen presentation by in vitro pulsing with tumor-derived RNA and reported the anti-tumor effect both in vitro and in mice [147]. Ex vivo DC loading, which achieves an oriented antigen presentation in vitro, has become a highly pursued delivery strategy of cancer vaccines to stimulate anti-tumor cellular immune responses. In 1999, Zhou et al. demonstrated tumor growth restriction and prolongation of survival time in a mouse–melanoma model by direct injection of glycoprotein 100 mRNA encapsulated in hemagglutinating virus of Japan (HVJ)-liposomes into the spleen, showing mRNA vaccines’ high potential against cancers [148].

### 5.1. mRNA Vaccines Against Infectious Diseases

Vaccines against infectious pathogens has always been the most effective way to prevent and limit infectious diseases, a classic example of which is the complete eradication of the smallpox virus. Unfortunately, traditional strategies of vaccines, such as non-live freeze-dried vaccines and live attenuated vaccines, underperform against some chronic or recurrent pathogenic infections with a long duration of disease such as AIDS and tuberculosis (TB). Traditional vaccines’ lack of adequate speed, owing to relatively slow process of development, would not be able to address outbreaks of virulent pathogens such as *Zaire ebolavirus*, Zika virus (ZIKV) and coronavirus.

mRNA vaccines against infectious diseases have made promising accomplishments and some products have entered human clinical trials (Table 2). Overall development steps of those vaccines are (1) constructing the core antigen-encoding mRNA sequence optimized or combined based on selected antigen(s) from the target pathogen; (2) trying and choosing a proper combination of mRNA construction type, adjuvants, carrier materials and the route of administration; (3) detecting in vivo expression of the encoded antigen and the level of elicited immune responses; (4) providing research and demonstrations of immune induction mechanisms. Here we have reviewed some recently published promising studies related to mRNA vaccine application trials.

#### 5.1.1. Influenza Virus

Influenza viruses have the characteristic of continuous evolution which makes them hard to be completely eradicated. The monoclonal antibody treatment targeting the conservative site of effector molecules of the influenza virus is commonly accepted as a highly specific and effective method against the virus [149]. mRNA vaccines encoding the conserved regions of influenza virus effector protein(s) are capable of provoking the generation of specific antibodies so that a better prevention or treatment effect, compared to conventional vaccines, is acheived. In addition, the rapid production process of mRNA vaccines makes them easier to stand out in preventing novel influenza virus. Current mRNA vaccines against influenza mostly use cationic lipids-based delivery systems to effectively deliver mRNA. The RNActive^®^ vaccine platform with the self-adjuvanting property give an impressive performance in trials of prevention of influenza, too [54]. Brazzoli et al. generated a novel oil-in-water CNE as the carrier for a SAM vaccine expressing influenza virus hemagglutinin (HA) antigen [150]. The vaccination was reported to effectively induce functional neutralizing antibody and HA-specific CD4^+^ Th1 cells and CD8^+^ cytotoxic T cells immune responses; it also defended a lethal influenza virus challenge in mice. Pardi et al. successfully elicited HA stalk-specific antibody response in mice, rabbits, and ferrets by immunization with nucleoside-modified non-replicating mRNA vaccine candidate encoding full-length influenza virus HA formulated in LNP [151]. This mRNA-LNP influenza vaccine partially overcome inhibition by the usage of maternal antibodies, and in turn induced a longer-lived and stronger immune protection in the mouse pups than a conventional influenza vaccine [152]. Feldman et al. reported phase I clinical trials of the first two non-replicating mRNA vaccines against influenza viruses (H10N8 and H7N9) encoding full-length HA respectively from H10N8 and H7N9 with a 1:20 mass ratio of mRNA to LNP [96]. Both vaccines used a LNP carrier that was first applied in mRNA vaccines against the Zika virus [96,98]; they were proved well tolerated by healthy adults and elicited potent humoral immune responses [96]. This research showed the potential of mRNA vaccines to address highly variable pathogens.

#### 5.1.2. HIV

AIDS, a chronic and life-threatening condition owing to the infection of HIV, has not yet found a truly effective and affordable way of cure since its discovery in 1981. Defeating HIV is a significant issue of research developing mRNA vaccines. At present, there are several mRNA vaccines for the treatment of AIDS in human clinical researches. Ex vivo loading of DC delivery systems seems to be a preferred delivery method which is normally used for cancer treatment. In infectious diseases, it is almost exclusively used for therapeutic research on AIDS, and is widely proved to safely cause antigen-specific CD4+ and CD8+ T cell immune response [21]. However, in 2016, Gandhi et al. reported disappointing results of a clinical trial for immunization of HIV-1-positive participants with autologous DCs transfected with mRNA encoding HIV-1 structural proteins Gag and Nef [153]. In that trial, merely transient and weak immune responses were detected, indicating the necessary improvement for the DC vaccination [153]. In such a way, delivery systems that can elicit strong antigen-specific T cell immune responses are getting more attention in AIDS treatment.

The cationic nanoparticle carrier is a promising delivery system with multiple diversity. Zhao et al. developed a PEI-stearic acid (PSA) copolymer-based self-assembled cationic nanomicelles which delivered non-replicating mRNA vaccine encoding HIV-1 Gag [109]. Their study initially showed the potential of PSA/mRNA nanomicelle vaccine strategy against HIV with acceptable carrier toxicity, efficient endosomal escape and translation of mRNA in DCs, and stimulated potent specific antibody secretion and pro-inflammatory cytokine expression [109]. Bogers et al. demonstrated a SAM vaccine encoding a HIV-1 clade C envelope glycoprotein delivered by a CNE system, including squalene, DOTAP, sorbitan trioleate and polysorbate, with a relatively mature preparation protocol [154]. Greater cellular immune responses and neutralizing antibody responses were induced by this HIV SAM vaccine instead of two other SAM vaccine modalities, the self-amplifying mRNA of which were encapsulated by a HIV recombinant envelope protein or in an engineered viral replicon particle [154].

HTI-TriMix, a combination of activation adjuvant TriMix and selected mRNA comprising of 16 conservative fragments from HIV-1 structural proteins—Gag, Pol, Vif, and Nef, is a new mRNA-based therapeutic vaccine candidate against HIV-1 [155]. It encodes strong activation signals and a potent HIV recombinant antigen. The preclinical results suggested an effective induction of mature DCs, antiviral cytokine secretion (especially IFN-γ) and T cell stimulation. Mice that were intranodally injected with HTI-TriMix generated potent antigen-specific cytotoxic T-cell responses [155]. By the end of 2019, phase I and phase IIa clinical trials of HTI-TriMix have been accomplished. In phase IIa, HIV-1-infected participants received three vaccinations at weeks 0, 2, and 4 detected through ultrasound-guided administration with an inguinal lymph node. Although HTI-TriMix showed good safety and tolerance, an unexpected start codon was unfortunately found upstream of the HTI recombinant antigen coding sequence which likely had a negative influence on HTI protein expression [69,156]. Future studies for corrected HTI are not yet certain. Taking into consideration of an additional translation process of mRNA vaccines, pre-testing of mRNA expression in vitro deserves our attention. Due to the limited understanding of HIV and the unclear pathogenesis, there are still many difficulties in the treatment of AIDS. Choosing proper antigen(s) and delivery system that can cause intense antigen-specific T cell immune response should be emphasized at mRNA vaccine design in the future. In addition, mRNA vaccines on AIDS prevention may also be a feasible field.

#### 5.1.3. Coronavirus

In the last 20 years, there have been three coronavirus infections (severe acute respiratory syndrome coronavirus (SARS-CoV), Middle East respiratory syndrome coronavirus (MERS-CoV) and (SARS-CoV-2)) globally, all leading to extreme health threats and tremendous economic loss without established therapies or vaccine treatment that would cure the illness. Of all the patents regarding vaccine types, most of them are related to SARS and MERS, only three patents have been focused on mRNA vaccines as of today [26]. In the face of the sudden new coronavirus epidemic, the speed of vaccine development determines the speed of life saving. Therefore, it is inevitable that mRNA vaccines with rapid product process will play an important role in the development of coronavirus vaccines.

COVID-19, caused by SARS-CoV-2 infection, has been spreading all over the world with over 23.51 million confirmed cases and over 810,000 deaths as of August 25, 2020 (data from World Health Organization). An effective vaccine is urgently needed. Lin et al. reported two non-replicating mRNA vaccines respectively encoding the receptor-binding domain of the spike protein and the virus-like particles (VLPs) of SARS-CoV-2; further optimization of antigen sequences, as well as safety and efficacy evaluations are underway [27]. Moderna first announced a mRNA vaccine candidate, mRNA-1273, against SARS-CoV-2, and officially began Phase I clinical trials for safety and immunogenicity evaluation on March 16, 2020. This vaccine encodes the spike (S) protein of SARS-CoV-2 in a prefusion stabilized form. According to the interim data announced on May 18, 2020, mRNA-1273 was shown generally safe and well tolerated; after two weeks following the second dose, with the vaccination dose as low as 25 µg, the levels of both binding antibodies and neutralizing antibodies in serum were at the levels detected in samples from people having recovered from COVID-19. Collaborative development of a new mRNA vaccine against SARS-CoV-2 has been announced by Sanofi Pasteur and Translate Bio on March 27, 2020. Pfizer and BioNTech announced the positive results of the ongoing phase I/II clinical trials of BNT162b1. It is a modified mRNA vaccine candidate formulated by LNP, encoding trimerized SARS-CoV-2 S protein receptor binding domain. Proper dose level of BNT162b1 was initially identified between 10 μg and 30 μg. After two doses of 10 μg and 30 μg of BNT162b1, mean titers of specific neutralizing antibodies were 1.8-fold and 2.8-fold, respectively, the specific neutralizing antibody of the convalescent [157].

#### 5.1.4. Other Viral Pathogens

CV7201, a prophylactic non-replicating mRNA candidate vaccine combined with protamine encoding rabies virus glycoprotein (RABV-G), completed phase I clinical trial in 2016 [90]. In pre-clinical trials, this vaccine elicited powerful functional antibody responses with a stable titer level up to one year and induced robust specific CD4^+^ and CD8^+^ T cells (higher CD4^+^ T cell induction than the induction by a licensed vaccine) when applied intradermally both in mice and pigs [158]. Although CV7201 was shown generally safe in phase I trial, the unstable administration-dependent functional antibody titer resulted in an unclear research outlook [90]. Subsequent new preclinical studies in 2019 reported an improved humoral and cell immune response using RABV-G mRNA packaged in LNP in both mice and nonhuman primates in comparison to the protamine formulated mRNA candidate; corresponding human clinical trials are being followed up [159].

In 2017, modified mRNA-LNP vaccines against ZIKV were reported in *Cell* and *Nature* respectively [97,98]. Pre-membrane (prM) protein and envelope (E), two ZIKV structural proteins, form prM-E heterotrimers when ZIKV buds invade the lumen of the endoplasmic reticulum. Richner et al. developed a LNP-encapsulated non-replicating mRNA vaccine encoding the human IgE signal sequence, which contained full-length prM and E genes (IgEsig-prM-E) [98]. Intramuscular inoculation of 2 μg of IgEsig-prM-E LNPs with a booster protected mice from severe ZIKV infection with remarkably high titers of neutralizing antibodies (>1/100,000 EC50) was detected [98]. Similarly, Pardi et al. demonstrated a low dose (50 μg) intradermal vaccination contained with mRNA-LNP complex encoding prM-E glycoproteins of ZIKV, which sufficiently protected non-human primates from a viral challenge [97].

Against Venezuelan equine encephalitis virus (VEEV), two synthetic CNE-encapsulated Venezuelan equine encephalitis SAM vaccine candidates, LAV-CNE (carrying the RNA genome of TC-83, a live-attenuated investigational vaccine strain) and IAV-CNE (carrying TC-83 viral genome with the capsid gene deleted), were designed to be capable of offering immune protection [160]. In inoculated mice, both vaccines induced robust virus-specific neutralizing antibodies and provided protection from wild-type VEEV aerosol challenge [160]. In addition, mRNA-based candidate vaccines have been developed and trialed against diverse viruses such as chikungunya virus, herpes simplex virus, human metapneumovirus and parainfluenza virus, all showing desirable development prospects [161,162,163].

It’s worth noting that Pepini et al. reported type I IFN, which played a critical role in antiviral responses and elicited by LNP-formulated SAM vaccine, could inhibit the expression of mRNA-encoded antigens in mice [164]. In line with this, Zhong et al. found that a naked ZIKV SAM vaccine encoding the ZIKV prM-E induced limited and unstably variable humoral immunity in wildtype mice when compared with robust response in IFNAR1 knockout mice [165]. Those researches suggest antiviral responses, especially type I IFN response, activated by SAM vaccination might have a negative effect on SAM-induced immune protection and optimization of SAM construction and administration should be considered.

#### 5.1.5. Bacterial Pathogens

Apart from viral antigens, only very few species of bacterial and parasitic antigens have been used in mRNA vaccine attempts, many of which still remain at the preclinical trial stage [166,167,168,169]. A wider variety of targeted antigens will represent more important issues for the next stage of mRNA vaccine development.

In 2017, Maruggi et al. designed two prophylactic SAM vaccines mixed with CNE encoding Streptolysin-O (SLOdm) from Group A (GAS) Streptococci and the pilus 2a backbone protein (BP-2a) from Group B (GBS) Streptococci, respectively [167]. Inoculated mice succeeded in producing a large amount of fully functional antibodies which could be significantly increased by booster, and survival rate was increased for GAS and GBS infections [167]. Among infectious diseases caused by a single pathogen, the TB caused by the bacterial pathogen *Mycobacterium tuberculosis* has been ranking first in fatality rate globally for a long time. Still, there is only one vaccine licensed against human TB: *Mycobacterium bovis* Bacillus Calmette–Guérin (BCG), an attenuated whole-cell vaccine which has been found severe limitations in numerous clinical trials [170,171]. MVA85A, a TB subunit vaccine expressing single antigen Ag85A, had no significant improved protection in phase IIb trial [172]. The letdown reminds us that, compared with the viral infections, bacterial infections tend to have more complicated stages with diverse characteristics of molecule expression, which are virtually impossible for single antigen to cover. If mRNA vaccines want to be applied further into the area of prevention and treatment of bacterial infection, more optimizations should be considered, including selection and recombination of various antigens or epitopes, periodic administration for different target antigens, and even direct addition of adjuvanted passive immune compositions (e.g., Kose et al. developed a chikungunya-against mRNA vaccine encoding neutralizing human monoclonal antibodies [173]).

We are all looking forward to the first successful mRNA vaccine product. Optimizing the primary and secondary structure of mRNA and choosing the appropriate delivery system according to the characteristics of different diseases are critical steps for better application of mRNA vaccines against various pathogens.

### 5.2. mRNA Cancer Vaccines

As our knowledge of tumor-specific antigens gradually deepens, people are now discussing more about the possibility of developing cancer vaccines [174,175]. Tumor-associated antigens (antigens preferentially expressed in cancerous cells and usually relevant to dysregulation and abnormal behaviors of cancerous cells) and tumor-specific neo-epitopes (small peptides derived from tumor-specific somatic mutation that are exposed to the surface of cancer cells and can be recognized by T cells) are now the core targets of mRNA cancer vaccines [18,176]. Considering the diversity and uncertainty of the cancerogenesis, cancer vaccines that are mainly therapeutic are now aimed to stimulate cellular immunity, which would potentially act as an effective cancer treatment [21,177]. There have been some clinical trials of hopeful candidates in progress (Table 3).

Sahin et al. pioneered the concept of “mutanome,” an overall detection and map of somatic mutations in individual tumors [18], the obtainment of which made personalized vaccination therapy possible and attractive with the help of next-generation sequencing technology [178,179]. Further, they designed procedures to develop personalized mRNA mutanome vaccines from mutanome identification, neo-epitopes prediction, and selection. This allowed mRNA vaccines to be unique for each patient. This strategy was firstly applied on melanoma patients with inspiring results achieved. By comparing tumor biopsies and normal blood cells via exome and RNA sequencing, researchers identified and selected ten mutations related to the human lymphocyte antigen (HLA) function per patient. Based on those mutations, core mRNA were synthesized and neo-epitope vaccines (≥eight doses) were percutaneously injected into inguinal lymph nodes [18]. Robust T cell responses against multiple neo-epitopes encoded by the vaccine were detected in all patients. With PD-1 blockade combination therapy, complete responses to vaccination were developed in some patients [18]. Clinical trials of similar mutanome-based mRNA vaccines against triple negative breast cancer are under way [180].

Co-transfecting mRNA encoding immune-regulation factors into DCs, normally by electroporation, to boost immune responses elicited by DCs-mRNA cancer vaccine has been an extensively studied subject [19,117,135]. TriMix can promote DC activation, CD4^+^ T cell phenotype shift, and cytotoxic T lymphocyte responses in numerous animal trials [68,155,181]. A joint therapy, which combined the vaccination of the DC-based mRNA, encoding TriMix and tumor antigens, plus ipilimumab (TriMixDC- MEL IPI), had been applied for advanced melanoma. It successfully induced potent tumor-associated antigen specific CD8^+^ T cell responses, demonstrating excellent therapeutic effects of the tumor-specific vaccine and immune checkpoint block agents [182]. Nevertheless, there existed undetectable response after in vitro T-cell stimulation in 3/15 patients, suggesting the necessity for a further study for mechanism of action about this issue. In addition, Reinhard et al. developed Chimeric antigen receptor (CAR)-T cells targeting regulated tight junction protein claudin 6 (CLDN6) supplemented by a liposomal CLDN6-encoding RNA vaccine which greatly boosted CAR-T cell stimulation and regression of large solid tumors in mice [183].

In view of significantly uneven distribution of various lymphocytes in the whole body and different locations and attributes of different primary tumors, it is essential to select the proper carrier and administration method for optimization of the mRNA vaccine effect [21,184]. General carrier systems and delivery routes are as stated above. For example, Jabulowsky et al. developed a RNA-lipoplex vaccine against melanoma [RNA(LIP)] which was injected intravenously to deliver mRNA steadily to APCs in whole body for antigen expression and presentation [185]. This vaccine is under clinical evaluation for safety and tolerance (NCT02410733). Direct intratumoral inoculation is notably an emerging method [21,181,186]. Shariati et al. pioneered the pressurized intraperitoneal aerosol chemotherapy (PIPAC) for delivering mRNA complexes and demonstrated that PIPAC is able to apply mRNA into peritoneal cavity in mice [187]. Besides, for optimization of carrier analysis and selection, a high-throughput approach to screen proper ionizable lipid-like materials as mRNA delivery vehicles were developed by Anderson et al. they constructed a combinatorial library of ionizable lipid-like materials using an isocyanide-mediated three-component reaction [105]; the screening standard was capable of facilitating in vivo mRNA delivery and providing effective and specific immune activation [105]. The best candidate chosen, heterocyclic lipids-mRNA vaccine, was further demonstrated to promote APCs maturation to stimulate potent immune responses via the intracellular stimulator of interferon genes pathway [105].

## 6. Discussion

As reviewed by Weissman et al., standardized in vitro good manufacturing practice of mRNA production is now accessible, while barriers still exist on synthesis of some uncommon sequences as well as salable and low-cost production for some reagents [21]. At the same time, capability of the long-term storage of mRNA vaccines with invariable activity should be emphasized. It had been reported early on that purified freeze-dried RNA in trehalose could maintain the activity up to 10 months at 4 °C, whose stability was comparable to conventional vaccines [188]. Phase I trial of mRNA-based rabies virus vaccine CV7201 demonstrated that it could be stored as a freeze-dried preparation at 5–25 °C for 3 years and at 40 °C for 6 months without obvious loss of activity [90]. In 2019, Coolen et al. developed a poly(lactic acid)-nanoparticle-based mRNA vaccine, mixed with amphipathic cationic peptides, which showed stable expression efficacy after storage at 4 °C for up to 7 days [189]. Although further investigations are needed to study the effects of storage of mRNA complexed with vector molecules under unfrozen condition, studies has suggested the potential of mRNA vaccines for cold-chain free transport and storage in the future [169].

What makes the mRNA vaccines a widely recognized form compared to conventional vaccines is the non-toxic production process, its short production time and chemical nature as ribonucleic acid, in line with safety and well-tolerance of mRNA vaccines shown in multiple clinical trials [14,69,77,90,96,156,162]. Various adverse symptoms, however, were still detected occasionally with unclear mechanism, emphasizing the importance of safety optimization [19,21]. Autoimmunity triggered by type I IFN responses are suggested to play a role in adverse physical reactions [164,165]. Other problems such as edema and coagulation due to excessive extracellular RNA and induction of anti-mRNA antibodies were also reported [22]; side effects owing to the vectors or administrating routes may also exist. Work on both safety assessment and investigations to mechanisms of the anti-vaccine response need to be moved forward.

Even though no mRNA vaccine product has been approved for marketing so far, development of specialized official product guidance of mRNA vaccines should be taken into consideration by medical authorities, particularly in view of the momentum and potential of this field where a remarkable number of relevant preclinical and clinical trials is active or completed.

The development of tools for material screening or characterization of mRNA-based complexes is of utmost importance to improve the stability and protein producing efficiency of mRNA vaccines. Constructing a combinatorial library of ionizable lipid-like materials is a promising strategy introduced above [105]. In 2018, Zhang et al. used the fluorescence correlation spectroscopy (FCS) to analyze the mRNA-based complex stability in buffer and biological fluid such as human serum and ascitic fluid [190]. Results have shown that strong mRNA binding of linear PEI would likely lead to a less efficient mRNA translation while a lipid-based carrier performed well in intracellular efficient release and subsequent translation of mRNA. Further, they applied FCS and single particle tracking to study the decay kinetics of mRNA with the half-life of mRNA in biological samples measured (~1–2 min) [191]. Single-molecule methods have a tempting application prospect of deep optimization for the construction of mRNA vaccines.

Theoretically, mRNA can be synthesized to express almost any protein antigens, which can provide a great flexibility for antigen design. For instance, a ZIKV vaccine from Richner et al. contains mRNA encoding a mutant antigen of ZIKV of which an immunodominant cross reactive epitope to dengue virus is deleted to minimize the induction of cross-reactive antibodies [98]. Nowadays, the application of artificial intelligence and deep learning lead to a huge progress in gene sequence-based prediction of protein structure [192,193]. The development of big data, meanwhile, immensely advances the improvement of algorithms for tumor antigen epitope prediction [194]. With the field of data mining further, it could become a reality to optimize existing antigens much better and design unprecedented antigens independent of natural genes. Optimization and personalization of mRNA vaccines will become a revolutionary milestone.

In conclusion, the mRNA vaccine is a versatile and powerful platform. Its successful development towards clinical translation will remarkably strengthen our ability to react to and control emerging communicable diseases, and prominently enrich our arsenal of treating classical and re-emerging communicable diseases and cancers from the perspective of stimulating self-immune responses. Further investigations for mechanisms of action of extracellular transportation and intracellular escape and gene expression of mRNA still deserve our efforts. Moreover, modularization of mRNA vaccine design and production targeting different application conditions seems to be a promising strategy for the clinical usage [169]. In the next 5 years, several critical clinical trials of mRNA vaccines are going to be completed (especially those against COVID-19). More extended human clinical experience will give us a more comprehensive insight into mRNA vaccine platform and various delivery systems.

From increasing productive capacity of mRNA and various carrier materials, to screening potential carrier molecules and adjuvants, to improving the composition and construction of vaccines, to arranging a corresponding route for administration, to optimizing the core encoding mRNA sequence and to demonstrating immune mechanisms of delivery and induction, the field of mRNA vaccines is still far from maturity, but its potential to be the preferred vaccine pattern has been fully shown.

## Figures and Tables

**Table 1:** Examples of mRNA delivery systems.

Delivery System (Delivery Methods/Materials)	Administration	Target Host	Disease(s)	Reference(s)
**Direct Injection**				
	Intradermal	Mice	--	[75,76]
	Intradermal	Human	Melanoma	[77]
	Intranodal	Mice	Cancer	[15]
	Intranodal	Mice	--	[67]
	Subcutaneous, intranasal, intravenous	Mice	--	[78]
	Subcutaneous tumors, intranodal	Mice	Cervical cancer	[79]
	Intranodal	Human	Melanoma	[18]
**Physical Delivery Methods**				
Electroporation				
	Intradermal	Mice	--	[80]
	--	Neurosphere	--	[81]
	--	DCs	Melanoma	[18]
Gene gun				
	--	Mice	Melanoma	[82]
	--	Mice	Epidermolysis bullosa	[83]
Sonophoresis				
	--	DCs	--	[84]
Microneedles				
	--	Mice	--	[85]
	Intradermal	Pig	--	[86]
**Ex Vivo Loading of DCs Delivery**				
	Subcutaneous	Mice	Different tumors	[84]
	Intradermal	Human	Acute myeloid leukemia	[87]
	--	Mice	Glioblastoma	[88]
**Protamine-Formulated Delivery**				
	Intradermal	Human	Melanoma	[14]
	Intradermal, Intranodal	Mice, ferret, pig	Cancer, infectious diseases	[54]
	Intradermal	Human	Prostate cancer	[89]
	Intradermal, intramuscular	Human	Rabies	[90]
	Intradermal	Human	NSCLC	[91]
	Intradermal	Human	NSCLC	[92]
**Lipid-Based Delivery**				
LNP	Intramuscular	Mice, rat	Respiratory syncytial virus infection	[17]
LNP	Intravenous, intraperitoneal, subcutaneous, intramuscular, intradermal, intratracheal	Mice	--	[93]
LNP	Intravenous	Human	Melanoma	[94]
LNP	Intramuscular	Human	H10N8 and H7N9	[95,96]
LNP	Intradermal, intravenous, subcutaneous	Mice, rhesus macaque	ZIKV	[97]
LNP	Intramuscular	Mice	ZIKV	[98]
**Lipid-Based Delivery**				
LNP	Intravenous	Human adipocyte, hepatocyte	Anemia	[99]
LNP	Nasal pumping	Mice	Cystic fibrosis	[100]
LNP	Intravenous	Rat, monkey	Anemia	[101]
LNP	Intravenous	Mice	Cancer	[102]
DOTAP/DOPE	Subcutaneous	Mice	AIDS	[61]
DOPE/DC-Cholesterol (2:1)	--	A549 Cells	--	[103]
DOTMA/DOPE or DOTMA/ cholesterol	Intravenous	Mice	--	[104]
Lipid library	--	DCs, HeLa cells	Melanoma	[105]
**Polymer-Based Delivery**				
PBAE	Subretinal injections	Mice	Retina diseases	[106]
PBAE, lipid-PEG	Intravenous	Mice	--	[107]
Poly(glycoamidoamine)	Intravenous	Mice	Anemia, myelodysplasia	[108]
PSA, PEI	Subcutaneous	Mice	AIDS	[109]
PEI-PEG	Intravenous	Mice	Pulmonary vascular disease	[110]
PEG[Glu(DET)]_2_	Subcutaneous	Mice	Muscle atrophy	[111]
hPBAEs	Inhalation	Mice	--	[112]
DEAE-Dextran	--	DCs	--	[113]
				
**Lipid and Polymer Hybrid**				
DOTMA, PLGA	--	DCs	--	[114]
LNP and polymer micelle	Intravenous	Mice	Ornithine transcarbamylase deficiency	[115]
CLAN (PEG-PLGA, PLGA, BHEM-cholesterol)	Intravenous	Mice, DCs	Lymphoma	[116]

**Abbreviations:** NSCLC, Non-small cell lung cancer; ZIKV, Zika virus; AIDS, acquired immune deficiency syndrome; DCs, dendritic cells; LNP, lipid nanoparticles (ionizable cationic lipid, PEG, cholesterol, phospholipids); PEG, polyethylene glycol; DOTAP, dioleoyl-3-trimethylammonium propane; DOPE, dioleoylphosphatidylethanolamine; DC-Cholesterol, 3β-[N-(N’,N’-dimethylaminoethane) carbamoyl]; DOTMA, N-[1-(2,3-dioleoyloxy)propyl]-N,N,N- trimethylammonium chloride; PBAE, poly(β-amino ester); PSA, polyethyleneimine-stearic acid; PEI, polyethylenimine; DEAE, diethylaminoethyl; hPBAEs, hyperbranched poly(beta amino esters); PEG[Glu(DET)]_2_, N-substituted polyethylene glycol-diblock-polyglutamide; PLGA, poly(lactic-co-glycolic acid); CLAN, cationic lipid-assisted nanoparticles; BHEM-cholesterol, N-bis(2-hydroxyethyl)-N-methyl-N-(2-cholesteryloxycarbonyl aminoethyl) ammonium bromide.

**Table 2:** Examples of mRNA vaccine clinical trials for infectious diseases.

Study Product	Antigen	Delivery Carrier	Administration	Phase	NCT Identifier	Status	Target
--	--	DCs	intradermal	Ⅰ/Ⅱ	NCT00833781	Completed	AIDS
iHIVARNA-01	HTI	DCs	inguinal intranodal	Ⅰ	NCT02413645	Completed	AIDS
iHIVARNA-01	HTI	DCs	intranasal	Ⅱ	NCT02888756	Terminated	AIDS
mRNA-1647/mRNA-1443	CMV associated antigens	--	--	Ⅰ	NCT03382405	Active, not recruiting	CMV infection
mRNA-1647	gB, pentamer complex	--	--	Ⅱ	NCT04232280	Recruiting	CMV infection
mRNA-1273	Spike protein	lipsome	intramuscular	Ⅰ	NCT04283461	Active, not recruiting	COVID-19
mRNA-1273	Spike protein	lipsome	--	Ⅱ	NCT04405076	Active, not recruiting	COVID-19
BNT162a1/BNT162b1/BNT162b2/BNT162c2	Spike protein	LNP	intramuscular	Ⅰ/Ⅱ	NCT04380701	Recruiting	COVID-19
BNT162a1/BNT162b1/BNT162b2/BNT162c2	Spike protein	LNP	intramuscular	Ⅰ/Ⅱ	NCT04368728	Recruiting	COVID-19
CVnCoV Vaccine	Spike protein	--	intramuscular	Ⅰ	NCT04449276	Recruiting	COVID-19
VAL-506440	H10N8 HA	LNP	intramuscular/intradermal	Ⅰ	NCT03076385	Completed	Influenza
VAL-339851	H7N9 HA	LNP	intramuscular	Ⅰ	NCT03345043	Active, not recruiting	Influenza
mRNA- 1653	hMPV, PIV3	--	--	Ⅰ	NCT03392389	Completed	hMPV infection
mRNA- 1653	hMPV, PIV3	--	--	Ⅰ	NCT04144348	Recruiting	hMPV infection
CV7201	Rabies virus glycoprotein	RNActive^®^	--	Ⅰ	NCT02241135	Completed	Rabies
CV7202	RABV-G protein antigens	--	intramuscular	Ⅰ	NCT03713086	Active, not recruiting	Rabies
mRNA- 1325	--	--	--	Ⅰ	NCT03014089	Completed	Zika virus
mRNA- 1893	Zika virus associated antigen	--	--	Ⅰ	NCT04064905	Recruiting	Zika virus

**Abbreviations:** HIV, human immunodeficiency virus; CMV, cytomegalovirus; gB, herpesvirus glycoprotein; HA, hemagglutinin; hMPV, human metapneumovirus; PIV3, parainfluenza virus 3; RABV-G, rabies virus glycoprotein; DCs, dendritic cells; LNP, lipid nanoparticles; AIDS, acquired immune deficiency syndrome; COVID-19, coronavirus disease 2019.

**Table 3:** Examples of mRNA vaccine clinical trials for cancers.

Study Product	Antigen	Delivery Carrier	Administration	Phase	NCT Identifier	Status	Target
--	WT1	DCs	intradermal	Ⅰ	NCT00834002	Completed	AML
--	WT1	DCs	intradermal	Ⅱ	NCT01686334	Recruiting	AML
--	Leukemia associated antigens, CMV antigen	DCs	intradermal	Ⅰ/Ⅱ	NCT01734304	Completed	AML
--	WT1	DCs	--	Ⅰ/Ⅱ	NCT03083054	Active, not recruiting	AML
GRNVAC1	hTERT, LAMP-1	DCs	--	Ⅱ	NCT00510133	Completed	AML
--	Leukemia associated antigens	DCs	--	Ⅰ	NCT00514189	Terminated	AML
--	--	DCs	--	Ⅰ	NCT02808416	Active, not recruiting	Brain metastases
--	CEA	DCs	intravenous/intradermal	Ⅰ/Ⅱ	NCT00228189	Completed	Colorectal cancer, liver metastases
--	MUC1, survivin	DCs	--	Ⅰ/Ⅱ	NCT02693236	Unknown *	Esophagus cancer
--	--	DCs	intradermal	Ⅰ/Ⅱ	NCT00846456	Completed	GBM
--	Human CMV pp65-LAMP, HIV-Gag	DCs	intradermal	Ⅱ	NCT03688178	Suspended	GBM
--	Human CMV pp65-LAMP	DCs	intradermal	Ⅱ	NCT02366728	Active, not recruiting	GBM
--	WT1	DCs	intradermal	Ⅰ/Ⅱ	NCT02649582	Recruiting	GBM
--	--	DCs	Intravenous/intradermal	Ⅰ	NCT02709616	Active, not recruiting	GBM
PerCellVac2	Glioma associated antigens	DCs	--	Ⅰ	NCT02808364	Active, not recruiting	GBM
DEN-STEM	hTERT, survivin, autologous tumor antigens	DCs	intradermal	Ⅱ/Ⅲ	NCT03548571	Recruiting	GBM
pp65 DC	pp65	DCs	subcutaneous	Ⅱ	NCT02465268	Recruiting	GBM
I-ATTAC	Human CMV pp65-LAMP	DCs	intradermal	Ⅱ	NCT03927222	Recruiting	GBM
--	CMV pp65-LAMP	DCs	intradermal	Ⅰ	NCT00639639	Active, not recruiting	GBM
--	WT1	DCs	intradermal	Ⅰ/Ⅱ	NCT01291420	Unknown *	GBM
--	Brain tumor stem cell specific antigens	DCs	intradermal	Ⅰ	NCT00890032	Completed	GBM
--	MiHA	DCs	intravenous	Ⅰ/Ⅱ	NCT02528682	Recruiting	Hematological malignancies
--	CMV pp65-LAMP	DCs	intradermal	Ⅰ	NCT00626483	Completed	Malignant neoplasms Brain
--	WT1	DCs	intradermal	Ⅰ/Ⅱ	NCT02649829	Recruiting	Malignant pleural mesothelioma
--	--	DCs	intradermal/intranasal	Ⅰ/Ⅱ	NCT01278940	Completed	Melanoma
--	gp100, tyrosinase	DCs	--	Ⅰ/Ⅱ	NCT00243529	Completed	Melanoma
--	Melan-A, Mage-A1, Mage-A3, survivin, gp100, tyrosinase	--	intradermal	Ⅰ/Ⅱ	NCT00204607	Completed	Melanoma
--	Melan-A, Mage-A1, Mage-A3, Survivin, gp100, tyrosinase	--	intradermal	Ⅰ/Ⅱ	NCT00204516	Completed	Melanoma
--	gp100, tyrosinase	DCs	intradermal/intravenous	Ⅰ/Ⅱ	NCT00940004	Completed	Melanoma
--	hTERT, survivin	DCs	--	Ⅰ/Ⅱ	NCT00961844	Terminated	Melanoma
--	hTERT, survivin, p53	DCs	intradermal	Ⅰ	NCT00978913	Completed	Melanoma
--	--	DCs	intravenous/intranasal	Ⅰ	NCT01066390	Completed	Melanoma
--	gp 100, tyrosinase	DCs	intranasal	Ⅰ/Ⅱ	NCT01530698	Completed	Melanoma
--	gp 100, tyrosinase	DCs	Intradermal/intravenous	Ⅱ	NCT02285413	Completed	Melanoma
--	TRP2	DCs	subcutaneous	Ⅰ	NCT01456104	Active, not recruiting	Melanoma
mRNA- 4157	multiple neoantigens	--	--	Ⅱ	NCT03897881	Recruiting	Melanoma
NCI-4650	--	--	intramuscular	Ⅰ/Ⅱ	NCT03480152	Terminated	Melanoma
--	CT7, Mage-A3, WT1	DCs	subcutaneous	Ⅰ	NCT01995708	Active, not recruiting	Multiple myeloma
CV9201	--	RNActive^®^	--	Ⅰ/Ⅱ	NCT00923312	Completed	NSCLC
DC-CIK	SOCS 1, MUC1, survivin	DCs	--	Ⅰ/Ⅱ	NCT02688686	Unknown *	NSCLC
BI 1361849	--	--	--	Ⅰ/Ⅱ	NCT03164772	Recruiting	NSCLC
DC-006 vaccine	hTERT, survivin	DCs	intradermal	Ⅰ/Ⅱ	NCT01334047	Terminated	Ovarian cancer
W_ova1 Vaccine	--	Liposome	intravenous	Ⅰ	NCT04163094	Recruiting	Ovarian cancer
--	TERT	DCs	--	Ⅰ	NCT01456065	Unknown *	Ovarian cancer
--	hTERT, survivin	DCs	--	Ⅰ/Ⅱ	NCT01197625	Active, not recruiting	Prostate cancer
--	NY-ESO-1, MUC1 PepTivator^®^	protamine and DCs	intranasal	Ⅱ	NCT02692976	Completed	Prostate cancer
--	hTERT, survivin, PSA, PAP	DCs	intradermal	Ⅱ	NCT01446731	Completed	Prostate cancer
CV9104	Prostate associated antigens	RNActive^®^	intradermal	Ⅱ	NCT02140138	Terminated	Prostate cancer
mRNA- 4157	multiple neoantigens	--	intramuscular	Ⅰ	NCT03313778	Recruiting	Solid tumors

* Studies that have passed their completion date and status have not been verified in more than two years. **Abbreviations:** WT1, Wilms’ Tumor-1; CMV, cytomegalovirus; hTERT, human telomerase reverse transcriptase; LAMP, lysosome-associated membrane protein; CEA, carcinoembryonic antigens; MUC1, tumor marker expressed by MUC1 gene; HIV, human immunodeficiency virus; Survivin, one of the apoptosis inhibitory protein family; pp65, 65K phosphoprotein; MiHA, minor histocompatibility antigens; gp100, glycoprotein 100; Melan-A, Melanoma antigen recognized by T cells; Mage-A1 and Mage-A3 and CT7, cancer testis antigen; TRP2, tyrosinase-related protein 2; SOCS 1, cytokine signaling 1; TERT, telomerase reverse transcriptase; NY-ESO-1, New York esophageal squamous cell carcinoma 1; PSA, prostate specific antigen; PAP, prostatic acid phosphatase; DCs, dendritic cells; AML, Acute myelocytic leukemia; GBM, Glioblastoma; NSCLC, Non-small cell lung cancer.

## Abbreviations

mRNA	messenger ribonucleic acid
DOTMA	N-[1-(2,3-dioleyloxy) propyl]-N,N,N-trimethylammonium chloride
TNF-α	tumor necrosis factor-α
IFN-α	interferon-α
COVID-19	coronavirus disease 2019
5′ cap	five-prime cap
5′ UTR	five-prime untranslated region
ORF	open reading frame
3′ UTR	three-prime untranslated region
eIF	eukaryotic translation initiation factor
ARCA	anti-reverse cap analogs
DCs	dendritic cells
PABP	Poly (A) binding protein
APCs	antigen-presenting cells
PRRs	pattern recognition receptors
TLR	Toll-like receptor
dsRNA	double-stranded RNA
RIG-I	Retinoic-acid-inducible gene I
RLRs	RIG-I-like receptors
MDA5	melanoma differentiation-associated 5
IFN	type I interferon
CD	cluster of differentiation
ssRNA	single-stranded RNA
PAMP	pathogen-associated molecular pattern
ISGs	IFN-stimulated genes
IFIT	IFN-inducible protein with tetratricoid repeats
OAS	2′-5′-oligoadenylate synthetase
CNE	cationic nanoemulsion
AIDS	acquired immune deficiency syndrome
HIV	human immunodeficiency virus
DOTAP	1,2-dioleoyl-3trimethylammonium-propane
DOPE	1,2-dioleoyl-sn-glycero-3-phosphoethanolamine
Th1	T-help 1 cell
NSCLC	Non-small cell lung cancer
ZIKV	Zika virus
LNP	lipid nanoparticles
PEG	polyethylene glycol
DC-Cholesterol	3β-[N-(N’,N’-dimethylaminoethane) carbamoyl]
PBAE	poly(β-amino ester)
PSA	polyethyleneimine-stearic acid
PEI	polyethylenimine
DEAE	diethylaminoethyl
hPBAEs	hyperbranched poly(beta amino esters)
PEG[Glu(DET)]2	N-substituted polyethylene glycol-diblock-polyglutamide
PLGA	poly(lactic-co-glycolic acid)
CLAN	cationic lipid-assisted nanoparticles
BHEM-cholesterol	N-bis(2-hydroxyethyl)-N-methyl-N-(2-cholesteryloxycarbonyl aminoethyl) ammonium bromide
MHC	major histocompatibility complex
CTLs	cytotoxic T-lymphocytes
siRNA	short interfering RNA
SAM	self-amplifying mRNA
gp100	glycoprotein 100
HVJ	hemagglutinating virus of Japan
TB	tuberculosis
CMV	cytomegalovirus
gB	herpesvirus glycoprotein
HA	hemagglutinin
hMPV	human metapneumovirus
PIV3	parainfluenza virus 3
RABV-G	rabies virus glycoprotein
SARS-CoV	severe acute respiratory syndrome coronavirus
MERS-CoV	Middle East respiratory syndrome coronavirus
S protein	spike protein
prM	pre-membrane
E	envelope
VEEV	Venezuelan equine encephalitis virus
GAS	Group A Streptococci
BCG	Mycobacterium bovis Bacillus Calmette–Guérin
WT1	Wilms’ Tumor-1
hTERT	human telomerase reverse transcriptase
LAMP	lysosome-associated membrane protein
CEA	carcinoembryonic antigens
MUC1	tumor marker expressed by MUC1 gene
Survivin	one of the apoptosis inhibitory protein family
pp65	65K phosphoprotein
MiHA	minor histocompatibility antigens
Melan-A	melanoma antigen recognized by T cells
TRP2	tyrosinase-related protein 2
SOCS 1	cytokine signaling 1
NY-ESO-1	New York esophageal squamous cell carcinoma 1
PAP	prostatic acid phosphatase
AML	acute myelocytic leukemia
GBM	glioblastoma
CAR	chimeric antigen receptor
CLDN6	claudin 6
PIPAC	pressurized intraperitoneal aerosol chemotherapy
FCS	fluorescence correlation spectroscopy
